# Time-Resolved and Tissue-Specific Systems Analysis of the Pathogenesis of Insulin Resistance

**DOI:** 10.1371/journal.pone.0008817

**Published:** 2010-01-21

**Authors:** Robert Kleemann, Marjan van Erk, Lars Verschuren, Anita M. van den Hoek, Maud Koek, Peter Y. Wielinga, Annie Jie, Linette Pellis, Ivana Bobeldijk-Pastorova, Thomas Kelder, Karin Toet, Suzan Wopereis, Nicole Cnubben, Chris Evelo, Ben van Ommen, Teake Kooistra

**Affiliations:** 1 Quality of Life, Vascular and Metabolic Disease, TNO, Leiden, The Netherlands; 2 Department of Vascular Surgery, Leiden University Medical Center, Leiden, The Netherlands; 3 Quality of Life, Physiological Genomics, TNO, Zeist, The Netherlands; 4 Top Institute Food and Nutrition, Wageningen, The Netherlands; 5 Department of Bioinformatics, Maastricht University, Maastricht, The Netherlands; 6 Quality of Life, Analytical Research, TNO, Zeist, The Netherlands; University of Las Palmas de Gran Canaria, Spain

## Abstract

**Background:**

The sequence of events leading to the development of insulin resistance (IR) as well as the underlying pathophysiological mechanisms are incompletely understood. As reductionist approaches have been largely unsuccessful in providing an understanding of the pathogenesis of IR, there is a need for an integrative, time-resolved approach to elucidate the development of the disease.

**Methodology/Principal Findings:**

Male ApoE3Leiden transgenic mice exhibiting a humanized lipid metabolism were fed a high-fat diet (HFD) for 0, 1, 6, 9, or 12 weeks. Development of IR was monitored in individual mice over time by performing glucose tolerance tests and measuring specific biomarkers in plasma, and hyperinsulinemic-euglycemic clamp analysis to assess IR in a tissue-specific manner. To elucidate the dynamics and tissue-specificity of metabolic and inflammatory processes key to IR development, a time-resolved systems analysis of gene expression and metabolite levels in liver, white adipose tissue (WAT), and muscle was performed. During HFD feeding, the mice became increasingly obese and showed a gradual increase in glucose intolerance. IR became first manifest in liver (week 6) and then in WAT (week 12), while skeletal muscle remained insulin-sensitive. Microarray analysis showed rapid upregulation of carbohydrate (only liver) and lipid metabolism genes (liver, WAT). Metabolomics revealed significant changes in the ratio of saturated to polyunsaturated fatty acids (liver, WAT, plasma) and in the concentrations of glucose, gluconeogenesis and Krebs cycle metabolites, and branched amino acids (liver). HFD evoked an early hepatic inflammatory response which then gradually declined to near baseline. By contrast, inflammation in WAT increased over time, reaching highest values in week 12. In skeletal muscle, carbohydrate metabolism, lipid metabolism, and inflammation was gradually suppressed with HFD.

**Conclusions/Significance:**

HFD-induced IR is a time- and tissue-dependent process that starts in liver and proceeds in WAT. IR development is paralleled by tissue-specific gene expression changes, metabolic adjustments, changes in lipid composition, and inflammatory responses in liver and WAT involving p65-NFkB and SOCS3. The alterations in skeletal muscle are largely opposite to those in liver and WAT.

## Introduction

Diabetes mellitus type 2 (DM2) is a metabolic disorder that is primarily characterized by insulin resistance (IR), relative insulin deficiency and hyperglycemia. DM2 is rapidly increasing in the developed world, and there is some evidence that this pattern will be followed in much of the rest of the world in the coming years [1].

IR is the condition in which regular amounts of insulin are inadequate to produce a normal insulin response in liver, adipose and muscle cells, and a key factor in the pathogenesis of DM2 [2]. The defects in insulin action in glucose metabolism include deficiencies in the ability of the hormone to suppress glucose production by the liver and to mediate glucose uptake and metabolism in insulin sensitive peripheral tissues (skeletal muscle and adipose tissue). The genetic and molecular bases for these reductions in insulin sensitivity are not fully understood.

IR is positively associated with visceral adiposity, i.e. a high degree of fatty tissue underneath the abdominal muscle wall [1]. Although epidemiological correlations are established, the cellular and molecular mechanisms that link obesity to IR are largely unknown. Growing evidence associates a chronic, subacute inflammatory state with the development of obesity and IR [1], [3]. Mediators of inflammation and the acute-phase response are positively associated with a risk of future IR/DM2 in humans [4] and can induce the disease in rodent models [5], [6], suggesting that low-grade inflammation precedes and triggers the development of IR/DM2. Indeed, proinflammatory cytokines such as TNFα, MCP-1, MIF can cause IR and anti-inflammatory medications may reverse it [5]–[9], and pathways involving the induction of nuclear factor-kB (NF-kB) or suppression of cytokine signaling (SOCS) proteins may be involved in the pathogenesis [8], [10]. An important unsolved issue is the origin of the inflammatory mediators and what stresses cause the activation of inflammatory pathways. There is ample evidence that the inflammatory response that emerges in the presence of obesity is triggered by metabolic dysregulation and resides predominantly in white adipose tissue (WAT), although other metabolically critical sites, particularly the liver, might also be involved [3]. Recent data from experimental models also indicate that metabolic stress is critical to the initiation and integration of this network of inflammatory pathways in obesity and DM2 [2], [3], but the understanding of the exact link between nutrient excess and the emerging inflammatory response is still incomplete.

Because studies in humans can never be as targeted as the experimental manipulations achievable in animal models, pathophysiological and mechanistic information is often obtained from studies in mice that are fed a high fat diet (HFD). A frequent limitation of these studies is that they are not time-resolved and that they, inherently to this set-up, provide information of a single time point or disease stage. Consequently, remarkably little is known about the line of events and tissue-specificity of HFD-induced IR and the dynamics of the changes in key metabolic processes.

To gain more insight into the disease process, we herein studied the association between excessive intake of dietary fat (causing metabolic dysregulation and metabolic stress) and the development of obesity and IR, using an integrative time-resolved approach. We systematically established the sequence of events leading to metabolic stress, tissue-specific inflammation, abdominal adiposity and organ-specific (liver, WAT, skeletal muscle) development of IR. HFD-treated ApoE*3-Leiden (E3L) mice were used because of the humanized lipoprotein metabolism and the translational value of this model for the human situation [11]. An important goal of the study was to identify – by combining transcriptomics with metabolomics and a comprehensive free fatty acid analysis – the molecular entities showing differential expression in liver or periphery (WAT, muscle) during initiation and progression of IR development. Particular emphasis was put on the biological processes and molecular networks that mediate metabolic dysregulation and that are associated with pathophysiological changes in liver and WAT, viz. the time course of changes in lipid and carbohydrate metabolism linked to inflammatory responses. This integrative approach provided solid evidence for and insight into the time-dependent, tissue-specific development of IR and the associated series of parallel and successive alterations in metabolic and inflammatory processes.

## Materials and Methods

### Animals and Diets

All animals received humane care according to the criteria outlined in the “Guide for the Care and Use of Laboratory Animals” prepared by the National Academy of Sciences and published by the National Institutes of Health (NIH). All animal experiments were approved by an independent institutional ethical committee on animal care and experimentation (Dierethische Commissie DEC, Zeist, The Netherlands). Male ApoE*3-Leiden transgenic (E3L) mice (n = 100 in total) were from TNO-BioSciences, Gaubius Laboratory, Leiden, The Netherlands. Mice were housed in groups (n≤4) wire-topped Macrolon cages and had access to water and diet *ad libitum*. Prior to high fat diet treatment animals received standard laboratory chow (Sniff R/M H chow, Research Diets, Uden, The Netherlands).

At the start of the experimental treatment period, E3L mice were 12 weeks of age. A tail blood sample was taken after 5 h of fasting in all animals. Then, animals were subjected to a glucose tolerance test (GTT) at t = −2.5 weeks, i.e. an intraperitoneal injection of glucose (2 g/kg body weight) to determine their individual ability to clear glucose. After a recovery period of 2.5 weeks (from -2.5 weeks to t = 0), animals were fed a high-fat diet (HFD) containing 24% w/w fat (beef tallow) equaling provision of 45% energy (diet 4031.05; Hope Farms, Woerden, The Netherlands). The HFD is composed of (in w/w) 24% fat (12% saturated, 10% monounsaturated and 1% polyunsaturated fatty acids, 21% protein and 35% carbohydrates (19% sugars); the diet composition of the manufacturer is provided as Table S1) for 12 weeks. Tail blood samples were collected after 5 h of fasting, and plasma was stored at −80°C until use. Groups of n = 15 animals (per time point) were subjected to a *second* i.p. GTT to assess the individual disease status at t = 0, and after 1 w, 6 w, 9 w and 12 w of HFD feeding. Three days after this second GTT, animals were fasted for 5 hours and euthanized with CO/CO_2_ to collect tissues (viz., liver, adipose tissue, and skeletal muscle) for transcriptomics, metabolomics and immunohistochemical analyses, i.e. Oil-Red O-staining for analysis of lipid droplets in cross-sections of liver and hematoxylin phloxine saffron (HPS)-staining for morphological analysis of adipocytes in WAT, essentially as described [5]. Tissues were snap-frozen immediately in liquid nitrogen, and stored at −80°C until use. Parallel to this time course experiment, additional groups of n = 6–9 animals each were subjected to hyperinsulinemic, euglycemic clamp analysis with radioactive tracers to assess tissue-specific insulin sensitivity at t = 0, 6 w and 12 w of HFD treatment.

### Analysis of Plasma Lipids, Lipoproteins, and Plasma Inflammation Markers

Total plasma cholesterol and triglyceride levels were measured after 5 hours of fasting, using kits No. 11489437 and 11488872 (Roche Diagnostics, Almere, The Netherlands), respectively, and NEFA with kit WAKO 999-75406 (FFA test NEFA-HR, Instruchemie, Delfzijl, The Netherlands). For lipoprotein profiles, pooled plasma was fractionated using an ÄKTA FPLC system (Pharmacia, Roosendaal, The Netherlands) [12]. The plasma levels of adiponectin, leptin, E-selectin, VCAM-1 (all R&D Systems Europe Ltd., Abington, UK), SAA (Biosource), vWF (in-house assay) and fibrinogen (in-house assay) were determined by ELISA as reported [12], [13].

### Glucose Tolerance Test and Hyperinsulinemic Euglycemic Clamp Analysis

Glucose tolerance tests were performed after a 5h-fasting period. Mice were injected intraperitoneally with glucose (2 g/kg body weight) and blood glucose levels were monitored for 120 minutes using a hand-held glucose analyzer (FreeStyle, Disectronics, Vianen, The Netherlands) at t = 0, 15 min, 30 min, 60 min and 120 min after glucose injection, essentially as described [5].

Hyperinsulinemic euglycemic clamp analysis was performed as described [5] (n = 6–9 animals per group). In short, after an overnight fast, mice were anesthetized with 6.25 mg/kg Acepromazine, (Sanofi sante animale, Libourne Cedex, France), 6.25 mg/kg Midazolam (Roche, Mijdrecht, The Netherlands) and 312.5 µg/kg Fentanyl (Janssen-Cilag, Tilburg, the Netherlands) and an infusion needle was placed in one of the tail veins. Subsequently, a bolus of insulin (4.1 mU Actrapid, Novo Nordisk, Chartres, France) was given, and a hyperinsulinemic euglycemic clamp was started with a continuous infusion of insulin (6.8 mU) and a variable amount of 12.5% D-glucose (in PBS) to maintain blood glucose levels at about 7.5 mmol/L. Blood samples were taken every 10 min and plasma glucose levels were measured using a hand-held glucose analyzer (FreeStyle, Disectronics, Vianen, The Netherlands) allowing to adapt glucose infusion rates accordingly [5].

### RNA Extraction for Microarray Analysis

Total RNA was extracted from individual tissues (liver, muscle, WAT (epidydimal)) using RNAzol (Campro Scientific, Veenendaal, The Netherlands) and glass beads [14]. The integrity of each RNA sample obtained was examined by Agilent Lab-on-a-chip technology using the RNA 6000 Nano LabChip kit and a bioanalyzer 2100 (Agilent Technologies, Amstelveen, The Netherlands). RNA preparations were considered suitable for array hybridization only if samples showed intact 18S and 28S rRNA bands, and displayed no chromosomal peaks or RNA degradation products (RNA Integrity Number >8.0) [15].

The One-Cycle Target Labeling and Control Reagent kit (Affymetrix #900493) and the protocols established by Affymetrix were used to prepare biotinylated cRNA (from 5μg of total RNA) for microarray hybridization (n = 8 for each tissue at each time point). The quality of intermediate products (i.e., biotin-labeled cRNA and fragmented cRNA) was again controlled using the RNA 6000 Nano Lab-on-a-chip and bioanalyzer 2100. Two muscle-tissue RNA samples (one t = 0 sample and one t = 6 weeks sample) did not pass the Quality Control check and were excluded from further analysis. Microarray analysis was carried out using an Affymetrix technology platform and Affymetrix GeneChip® mouse full genome 430 2.0 arrays (45,037 probe sets; 34,000 mouse genes). Briefly, fragmented cRNA was mixed with spiked controls, applied to Affymetrix Test chips, and good quality samples were then used to hybridize with murine GeneChip® 430 2.0 arrays. The hybridization, probe array washing and staining, and washing procedures were executed as described [15], and probe arrays were scanned with a Hewlett-Packard Gene Array Scanner (Leiden Genome Technology Center, Leiden, The Netherlands).

### Gene Expression Data Analysis

Quality control of microarray data was performed using BioConductor packages (including simpleaffy and affyplm, through the NuGO pipeline that is available as a Genepattern procedure on http://nbx2.nugo.org [16].

Two microarrays from WAT (week 1 and week 12) and one microarray from skeletal muscle (week 6) did not pass quality control criteria. The final microarray dataset consisted of 40 samples from liver, 38 samples from WAT and 37 samples from muscle according to procedures developed for this purpose [16]. Microarray data have been submitted to ArrayExpress (http://www.ebi.ac.uk/microarray-as/ae/).

The present study represents also one of the core studies of NuGO. The transcriptomics datasets are currently used by other researchers as announced recently (e.g. follow-up studies analyzing the various adipose tissue depots) [17].

For each tissue, raw signal intensities (from CEL files) were normalized using the GCRMA algorithm (gc-rma slow). Probesets were remapped and annotated into Entrez gene-ids using the custom MBNI CDF-file, version 9.0.1. This resulted in expression values for 16297 genes, represented by unique Entrez gene-ids. Genes were filtered on expression value above 5 in at least 4 samples, resulting in a set of 12251 genes for liver, 12492 genes for WAT and 10776 genes for muscle. Expression data were log-transformed (base 2). In muscle a batch effect of the two different labeling days was observed and a batch correction was performed.

Expression values of individual samples at weeks 1, 6, 9 and 12 were corrected for mean expression in samples of week 0, resulting in 2log ratios of expression compared to mean expression at week 0. T-profiler was used to assess enrichment of functional groups of genes (based on Gene Ontology) for all samples [18]. For each tissue, enriched functional groups were selected based on significant enrichment (t-score >|4|) in at least 5 samples of at least one time point. Next, a union list of enriched functional groups in the different tissues was submitted to hierarchical clustering (Genepattern, Broad Institute, MIT, USA). PathVisio [19] was used to visualize biological processes modulated by HFD in WAT and liver. Networks representing biological processes and their genes were generated based on pathways from the Kegg (www.genome.jp/kegg) and WikiPathways (www.wikipathways.org) pathway archives [20] (Version Feb. 2009) and visualized using Cytoscape and the VistaClara plugin [21]. Nodes in the network represent either a biological process or a gene. An edge is present between a process-node and gene-node when the gene plays a role in the biological process. The nodes representing genes that had no significant change (ANOVA q<0.01) in expression were filtered out. Thus connections between pathways only consist of genes that are present in both pathways and that showed a significant change over time. The size of nodes representing a biological process was scaled by a z-score representing enrichment of significant genes (ANOVA q<0.01).

Microarray gene expression data were confirmed by quantitative real-time PCR for a selection of genes using established protocols and primer/probe sets [14], [15].

### Analysis of Free Fatty Acids

5 µl of plasma or 5 mg lyophilized WAT or 5 mg lyophilized liver were used for a basic analysis of free fatty acids. Plasma and tissue homogenates were prepared as described [22] with the change that isopropanol instead of methanol was used for extraction of lipids. Analysis of the fatty acids extracts was according to the method reported by Pettinella and coworkers[23] which involves derivatization of fatty acids with quaternary ammonium, allowing an analysis of fatty acid derivatives in the positive mode and the discrimination of n-3 and n-6 fatty acids.

Measurements were carried out using a ThermoElectron LTQ-Orbitrap (LC-MS), equipped with a ThermoElectron Accela UHPLC with autosampler and column-oven (ThermoElectron, Breda, The Netherlands). 1.5 µL of the derivatized extract (maintained at 20°C in the autosampler) was injected onto a C8 column (Waters Acquity C8 1.7 µm particle size, 2.1 mm×100 mm). Biomolecules were separated at a flow-rate of 500 µL/min using a acetonitrile–aqueous formic acid gradient. Detection was performed using electrospray in the positive mode and data were processed using the LC Quan software (ThermoElectron, Breda, The Netherlands) for peak integration, calibration and quantification following the protocol of the manufacturer.

### Metabolomics Analysis

To analyze metabolite changes in liver, a recently developed GCxGC-MS-based method was followed [24]. In short, for sample preparation, the liver samples were freeze-dried overnight and homogenized. 10 mg aliquots of the liver samples were weighed and placed inside a two ml Eppendorf tube. After addition of 10 µl of IS mix 1 and 500 µl of methanol/water 4∶1 v/v, all samples were sonicated for 30 min and subsequently centrifuged for 10 min at 10000 rpm. The supernatants were transferred to autosampler vials and subsequently dried under nitrogen flow. Then 30 µl ethoxyamine hydrochloride was added and the samples were oximated for 90 min at 40°C on a tube roller mixer placed inside a stove. Subsequently, 100 µl of MSTFA was added and samples were silylated for 50 min at 40°C on a tube roller mixer. Finally the samples were centrifuged for 20 min at 3500 rpm prior to injection to remove debris.

The GCxGC-MS measurements were performed as described [24]. Briefly, derivatized samples were analyzed with an Agilent 6890 gas chromatograph fitted with a dual stage, four jet (two liquid nitrogen cooled and two hot gas jets) cryogenic modulator and a secondary oven (LECO, Mönchengladbach, Germany), and coupled to a time-of-flight mass spectrometer (Pegasus III, LECO). The configuration of the first (1D) and second dimension (2D) column and the method parameters were optimized [24]. A 30 m×0.25 mm I.D.×0.25 µm forte BPX-50 column (SGE Europe, Milton Keynes, UK) was used as the first dimension column and a 2 m×0.32 mm I.D.×0.25 µm forte BPX5 column (SGE Europe) as the second dimension column.

1-µl aliquots of the derivatized extracts were injected using PTV-injection (Gerstel CIS4 injector) in the splitless mode. The temperature of the PTV was 70°C during injection, and 0.6 min after injection the temperature was raised to 300°C at a rate of 2°C per second and held at 300°C for 20 minutes. The initial GC-oven temperature was 70°C, 3 min after injection the temperature was raised to 300°C with a rate of 5 °C/min and held at 300°C for 10 min. The temperature offset of the secondary oven and modulator compared to the GC oven were set at +30°C and +40°C, respectively. The modulation time was 6 seconds, with the hot pulse time set at 1 second. Helium was used as carrier gas and the analyses were carried out in constant pressure mode at 300 kPa. The MS transfer line was set at 325 °C and the ion source temperature was kept at 280°C. The detector voltage was set at 1600 V and the data acquisition rate was 75 Hz. The Chemstation software (Version E02.00.493, Agilent Technologies) was used for processing of the data. A target table was constructed using an in-house library of over 400 metabolites containing their spectra and retention times.

### Western Blot Analysis

Tissue extracts from liver, WAT and skeletal muscle were prepared in presence of proteinase inhibitors[25] and Western blot analyses was performed essentially as described previously[26] using antibodies specific for mouse SOCS3 (rabbit anti-SOCS3; ab3693; Abcam, Cambridge, UK) and PTP1B (rabbit anti-PTP1B; ab52650; Abcam, Cambridge, UK) and β-actin (anti-β-actin; sc-1615; Santa Cruz Biotechnology, Heerhugowaard, The Netherlands) for standardization of protein expression levels [27], [28].

Secondary antibodies were obtained from Santa Cruz Biotechnology and Pierce (Etten-Leur, The Netherlands) and immunoblots were visualized and quantified using the Super Signal West Dura Extended Duration Substrate (Perbio Science, Etten-Leur, The Netherlands), LabWorks 4.6 software and a luminescent image workstation (UVP, Cambridge, UK) [28]. SOCS3 and PTP1B protein expression data are reported as band intensity of immunostaining values (arbitrary units) as described by Guerra *et al* [29].

### Analysis of NF-κB Activity in Liver

To determine active p65-NF-κB activity in livers, tissue homogenates were prepared using the Nuclear Extract Kit (no. 40010, Active Motif, Rixensart, Belgium) and the protein content of the samples was determined[30] by the method of Bradford. Equal amounts of protein were used in the NF-κB TransAM transcription factor assay kit no. 40097 (Active Motif, Rixensart, Belgium). The assays were performed according to the manufacturer's instructions. Briefly, the amount of active transcription factor present in each sample was determined by measuring the binding of the active transcription factor to a consensus sequence in the presence of either a competitive or a mutated (non-competitive) oligonucleotide to correct for non-specific binding. Data are presented as relative units.

### Biochemical Analyses in Tissue Hepatic Homogenates

To determine the hepatic triglyceride content, liver biopsies were homogenized and the protein content was determined. 2 µg of cholesterol acetate was added as an internal standard. Lipids were extracted according to the Bligh and Dyer method [31]. The neutral lipids were separated by high performance thin layer chromatography on silica-gel-60 pre-coated plates and lipids were quantified by scanning the plates with a Hewlett Packard Scanjet 4c and subsequent computerized analysis of the density areas [14].

### Statistical Analysis

Statistical analysis was performed using Graphpad Prism 4.0 and SPSS 17.0. Plasma parameters (triglycerides, FFA, fibrinogen, SAA, VCAM-1, E-selectin, vWF, adiponectin) and body weight were determined in the group of mice (n = 15) that was sacrificed after 12 weeks of diet-feeding. Parameters requiring large volumes of plasma/serum for their quantification (cholesterol, ketone bodies, ALAT, leptin, glucose, insulin) were determined in plasma/serum collected at sacrifice. Changes over time (versus baseline t = 0) were statistically analyzed by with One-way ANOVA (repeated measures for within subject samples) and LSD post-hoc, unless stated otherwise.

## Results

### Effect of HFD Feeding on Adiposity and Plasma Lipids

Groups (n = 15 animals each) of E3L mice were fed HFD for 12 weeks. Food intake, body weight, plasma lipids and circulating tissue-specific inflammation markers were analyzed over time. Table 1 provides an overview of the baseline (t = 0), intermediate (t = 6 w) and end point (t = 12 w) levels.

**Table 1:** Body weight, plasma lipids and inflammation markers over time.

	t = 0	t = 6 w	t = 12 w
Body weight [g]	29.9±0.5	35.4±1.0***	37.5±1.1***
Cholesterol [mM]	2.15±0.23	5.29±0.35***	5.96±0.40***
Triglyceride [mM]	1.37±0.08	0.93±0.04***	0.97±0.04***
NEFAs [mM]	1.00±0.05	0.84±0.03*	1.08±0.04
Ketone bodies [mM]	0.47±0.05	0.48±0.04	0.26±0.02***
SAA [µg/ml] (L; A)	0.10±0.10	0.47±0.27	1.40±0.36**
Fibrinogen [mg/mL] (L)	2.29±0.16	2.89±0.18	3.94±0.34***
ALAT [U/mL] (L)	114±26	82±14	99±22
VCAM-1 [µg/ml] (V)	2.54±0.08	3.39±0.08***	3.50±0.20***
E-selectin [ng/mL] (V)	131.5±7.4	160.3±17.6*	153.9±10.0
vWF [% of pool] (V)	189±22	304±38	417±58***
Leptin [ng/ml] (A)	0.10±0.09	2.22±0.73	8.44±1.32***
Adiponectin [µg/mL] (A)	7.47±0.48	9.03±0.39*	9.44±0.59**

Lipids and inflammation markers determined in EDTA plasma obtained by tail bleeding after 5 h of fasting. Values of the time points t = 0, 6 and 12 weeks are shown. Letters in brackets indicate the main origin of the inflammation markers: liver (L), adipose tissue (A) or vasculature (V). Values are means ± SEM. Significance of post-hoc analysis is indicated * P<0.05, **P<0.01, ***P<0.001 (as compared to the starting level at t = 0).

There was a gradual increase in body weight, resulting in a significant difference in body weight between t = 0 (29.9±0.5 g) and t = 6 w (35.4±1.0 g; P<0.001). Body weight at the end of the treatment period was 37.5±1.1 g. The average food intake per mouse was 3.9 g per day.

**Figure 1:**
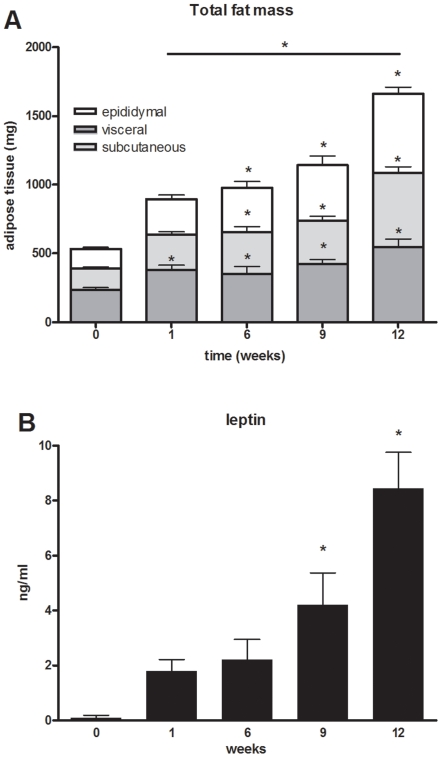
Fat mass over time. (**A**) Bars represent total fat mass subdivided into the epididymal, subcutaneous and visceral adipose tissue depots. Groups of n = 15 animals were sacrificed at the time points indicated to determine adipose mass of the various depots. Data are presented as means ± SEM. *P<0.05 indicates significance compared to t = 0 for each type of adipose tissue depot or the total fat mass. (**B**) Plasma leptin levels over time presented as means±SEM. *P<0.01 compared to t = 0.

Analysis of the mass of subcutaneous, visceral and epididymal fat pads of mice sacrificed at t = 0 and in weeks 1, 6, 9 and 12 of HFD feeding revealed a marked increase in adipose mass over time for all three fat depots (Figure 1A). The epididymal fat mass increased gradually over time, while the subcutaneous and visceral depots mainly increased in the first week and in the period between week 9 and 12.

The circulating leptin levels, which generally reflect adipose mass and differentiation [32], paralleled the increases in fat and body weight gain: leptin levels were very low at t = 0 (0.1±0.09 ng/ml) and increased strongly over time, reaching a concentration of 8.4±1.3 ng/mL (P<0.01) in week 12 (Figure 1B). Another adipokine, adiponectin, increased slightly but significantly in the first 6 weeks (from 7.47±0.48 µg/mL to 9.03±0.39 µg/mL, P<0.05), and remained at this elevated level from then on (Table 1).

Baseline plasma cholesterol levels (2.15±0.23 mM) increased rapidly in response to HFD feeding to 5.5±0.32 mM (P<0.05) in week 1, and remained at this elevated level until the end of the study (5.96±0.40 mM in week 12). Plasma triglyceride decreased slightly within one week from 1.37±0.08 mM to 1.00±0.06 mM (P<0.01) and then stayed at this level (0.97±0.04 mM in week 12; Table 1).

The plasma concentration of total free non-esterified fatty acids (NEFAs) was not markedly affected: the NEFAs level in week 12 (1.08±0.04 mM) was comparable to the baseline level (1.00±0.05 mM; Table 1). Plasma concentrations of ketone bodies did not change until week 9, but dropped markedly between week 9 and 12 (from 0.40±0.08 mM to 0.26±0.02 mM) pointing to decreased β-oxidation of fatty acids.

### HFD Feeding Alters Lipid Composition of Free Fatty Acids

**Figure 2:**
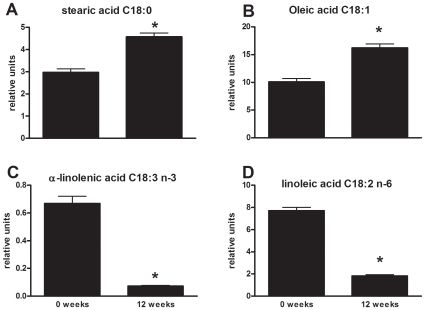
HFD feeding alters the relative amount of saturated, monounsaturated and polyunsaturated fatty acids. Free fatty acid analysis of plasma. Representative fatty acids of the C18 category (stearic acid, oleic acid, alpha-linolenic acid and linoleic acid) are shown. Data presented are relative amounts and values are means ± SEM. *P<0.001.

We next assessed whether HFD feeding may induce changes in the type of circulating fatty acids and thus may alter plasma lipid composition. Analysis of saturated, monounsaturated and polyunsaturated fatty acids by LC/MS revealed an important change in their relative amounts during HFD treatment (Figure 2 and Table S2). Stearic acid, a saturated fatty acid, and the monounsaturated fatty acids oleic and eicosenic acid significantly increased over time, while n-3 polyunsaturated fatty acids (PUFAs) such as alpha-linolenic acid, stearidonic acid, eicosatetraenoic acid and eicosapentaenoic acid (EPA), all of which considered to be beneficial for health, consistently decreased with HFD feeding. Also, the plasma concentration of several n-6 PUFAs, including linoleic acid, gamma-linolenic acid and adrenic acid, significantly and consistently declined during the 12 weeks HFD period (Table S2).

### HFD Feeding Induces Chronic Low-Grade Inflammation

The plasma levels of the liver-derived inflammation markers serum amyloid A (SAA) and fibrinogen gradually increased during the study period. SAA levels were very low at t = 0 (0.1±0.1 µg/mL) and reached concentrations of 1.4±0.4 µg/mL in week 12 (P<0.01), whereas fibrinogen concentrations increased from 2.3±0.2 mg/mL to 3.9±0.3 mg/mL during the same period (P<0.001), with most pronounced increases occurring after week 6 (Table 1). Of note, SAA and fibrinogen transiently peaked in week 1 (3.3±1.7 µg/mL) and week 3 (3.5±0.3 mg/mL), respectively, indicating an acute-type inflammatory response to HFD (data not shown). Plasma activity levels of ALAT, a marker of liver damage, did not change and remained at a constant level (Table 1).

VCAM-1 and E-selectin, markers for vascular inflammation and endothelial dysfunction, responded rapidly to HFD feeding and reached maximal plasma levels within 3 weeks: VCAM-1 increased from 2.5±0.1 µg/mL at t = 0 to 3.4±0.2 µg/mL in week 3 and E-selectin rose from 132±7 ng/mL at t = 0 to 158±6 ng/mL in week 3. The vascular endothelium activation marker, von Willebrand factor (vWF) increased continuously during HFD treatment, from 189±22% at t = 0 to 417±58% in week 12 (P<0.001; Table 1).

Together, HFD feeding caused metabolic as well as inflammatory changes over time as reflected by dyslipidemia, pronounced marked changes in fatty acid composition, and elevated levels of circulating inflammation markers.

### Effect of HFD on Metabolic Parameters and Glucose Tolerance

**Figure 3:**
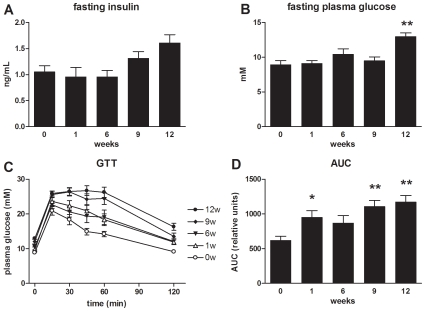
Effect of HFD feeding on fasting plasma insulin and glucose levels and glucose tolerance. Plasma insulin (**A**) and glucose (**B**) levels determined after a 5 hr fasting period. A glucose tolerance test (GTT) (**C**) was performed 2.5 weeks prior to inclusion into the study (not shown) and each animal underwent a second GTT, in week 0, 1, 6, 9, and 12 of HFD feeding (n = 15 each). After a 5 h fast, animals received 2 g glucose/kg body weight (i.p.). Glucose was recorded at the time points indicated and the area under the curve (AUC) (**D**) was calculated. Values are means ± SEM. **P<0.01; *P<0.05

Fasting plasma insulin and glucose levels were determined after a 5-h fasting period at the time points specified in Figure 3A/B. Insulin levels did not significantly change over the first 6 weeks and remained close to the t = 0 value of 1.1±0.4 mM; then, plasma insulin started to increase up to 1.3±0.2 mM in week 9 and 1.6±0.2 mM (P<0.05) in week 12. A similar, but delayed, pattern was seen for glucose: fasting plasma glucose levels did not significantly change between t = 0 (8.9±0.6 mM) and week 9 (9.5±0.6 mM), but increased significantly thereafter, reaching 13.0±0.5 mM (P<0.01) at 12 weeks of HFD. Fasting insulin and glucose concentrations were used to calculate the Homeostatic Model Assessment of Insulin Resistance (HOMA) index. HOMA remained unchanged until week 6 and increased then gradually (2.1-fold increase in week 12; P<0.01).

Glucose tolerance tests (GTT) were performed in each animal prior to inclusion into the study (at t = −2.5 weeks; data not shown) and 3 days before sacrifice (i.e. at 0, 1, 6, 9, 12 weeks of HFD feeding; Figure 3C). Prior to HFD treatment, mice were able to fully clear a bolus of glucose within 120 min (Figure 3C). This ability was lost already after 1 week of HFD feeding, and mice developed increasing glucose intolerance with prolonged HFD feeding. In accordance herewith, the area under the curve (AUC) increased over time and was significantly higher compared to t = 0 at all time points analyzed, except week 6 (Figure 3D).

### Time Course of Insulin Resistance (IR) Development in Liver, WAT, and Muscle by Clamp Analysis

**Figure 4:**
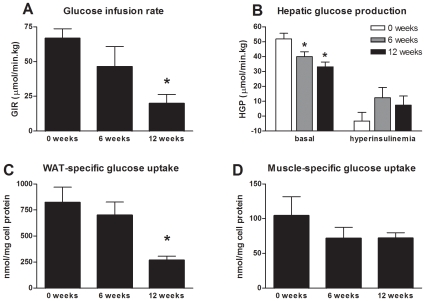
Glucose tolerance test and hyperinsulinemic, euglycemic clamp analysis. In a parallel, independent animal experiment, HFD-fed mice were subjected to hyperinsulinemic, euglycemic clamp analysis. (**A**) shows the glucose infusion rate (GIR) under hyperinsulinemic conditions. Radioactive tracers allowed determining hepatic glucose production rate (HGP) (**B**) as well as the tissue-specific uptake of glucose into WAT (**C**) and skeletal muscle (**D**). ‘Basal’ indicates prior to hyperinsulinemic clamp conditions and ‘hyper’ indicates hyperinsulinemic clamp conditions. Values are means ± SEM. *P<0.05 vs. t = 0.

To determine when and to what extent liver, adipose and muscle tissues become insulin insensitive, hyperinsulinemic-euglycemic clamp analysis was performed at t = 0, 6 and 12 weeks. The glucose infusion rate (GIR), a measure of whole body insulin sensitivity, was determined first. Consistent with a progressive development of IR over time, the GIR tended (P = 0.13) to be lower in week 6 when compared to t = 0 (Figure 4A) and became significantly reduced in week 12 (P<0.05).

The use of radioactive glucose tracers allowed us to determine the sequence of IR development in liver, WAT and muscle by analyzing the ability of insulin to shut down endogenous hepatic glucose production (HGP) and to stimulate the glucose uptake into adipose tissue and skeletal muscle.

HFD feeding alone reduced baseline endogenous HGP significantly in week 6 and 12 (Figure 4B; left panel). The application of hyperinsulinemic conditions almost fully shut down HGP at t = 0 demonstrating that the liver was insulin sensitive prior to HFD feeding (Figure 4B; right panel). After 6 and 12 weeks of HFD feeding the inhibitory effect of insulin became markedly weaker, clearly indicating a decreased insulin sensitivity of the liver at these time points. Consistent with this, the hepatic insulin sensitivity index (HISI) calculated from the equation HISI = (HGPbasal - HGPhyper)/HGPbasal was markedly reduced (by 40%; P<0.05) in week 6, pointing to hepatic IR.

At t = 0, the glucose uptake into WAT and muscle was 820±145 nmol/mg tissue protein and 105±27 nmol/mg tissue protein, respectively (Figures 4C and D). 6 weeks of HFD feeding not significantly affected glucose uptake into WAT and muscle. After 12 weeks however, glucose uptake into WAT, but not muscle, was strongly and significantly reduced (270±37 nmol/mg; P<0.05), clearly indicating that this tissue had become IR, notably at a later time point than liver.

### Time-Resolved and Tissue-Specific Microarray Analysis of Metabolic Processes

To gain further insight into the time course and metabolic processes underlying IR evolution, high quality RNA was prepared from liver, WAT and skeletal muscle and subjected to microarray analysis.

**Figure 5:**
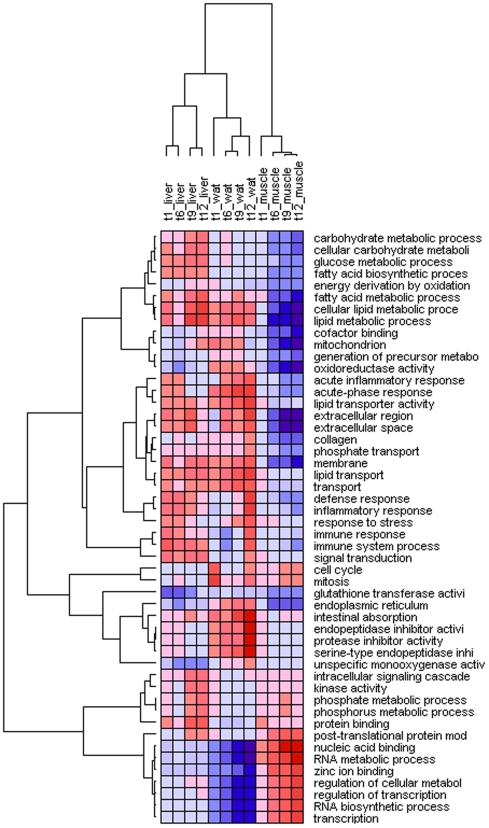
Time-resolved pathway analysis for liver, WAT and muscle. Hierarchically clustered heat map of enriched functional groups of genes (Gene Ontology). Shown are functional groups of genes that are significantly enriched in at least one time point and tissue. Clustering analysis (Pearson correlation, complete linkage) was performed on average t-values for each time point. Red (blue) indicates up-regulation (down-regulation) of a functional group and a positive (negative) t-value. The color intensity reflects the magnitude of a change. Columns represent the different tissues over time and rows represent the functional clusters.

Microarray data were analyzed by hierarchical clustering of enriched functional groups of genes (based on Gene Ontology) and the major results are graphically illustrated in a heat map (Figure 5). HFD elicited distinct changes in gene expression in liver, WAT and muscle of E3L mice over time, with most changes in liver and WAT being increases and those in skeletal muscles being decreases. Typically, in the early stages, the expression of genes is often only marginally modulated. With time, particularly when glucose intolerance and adipose mass proceed and other exacerbating factors may come into play, gene expression profiles become categorically increased in liver and WAT while they decreased in skeletal muscle.

The liver is a key player in the control of whole body energy metabolism by its ability to synthesize, oxidize, store, and distribute the major sources of energy, glucose and fatty acids. Accordingly, after starting the HFD feeding, the liver immediately responds by up-regulating the expression of genes involved in glucose metabolic processes, (cellular) carbohydrate metabolic processes, fatty acid biosynthetic and metabolic processes, (cellular) lipid metabolic processes, and lipid transport, while in WAT only the expression of genes involved in (cellular) lipid metabolic processes and lipid transport increase (Figure 5 and Figure S1). Of note, fatty acid biosynthesis is upregulated in liver and downregulated in WAT, while fatty acid metabolism is upregulated in both tissues, with WAT responding less strongly.

**Figure 6:**
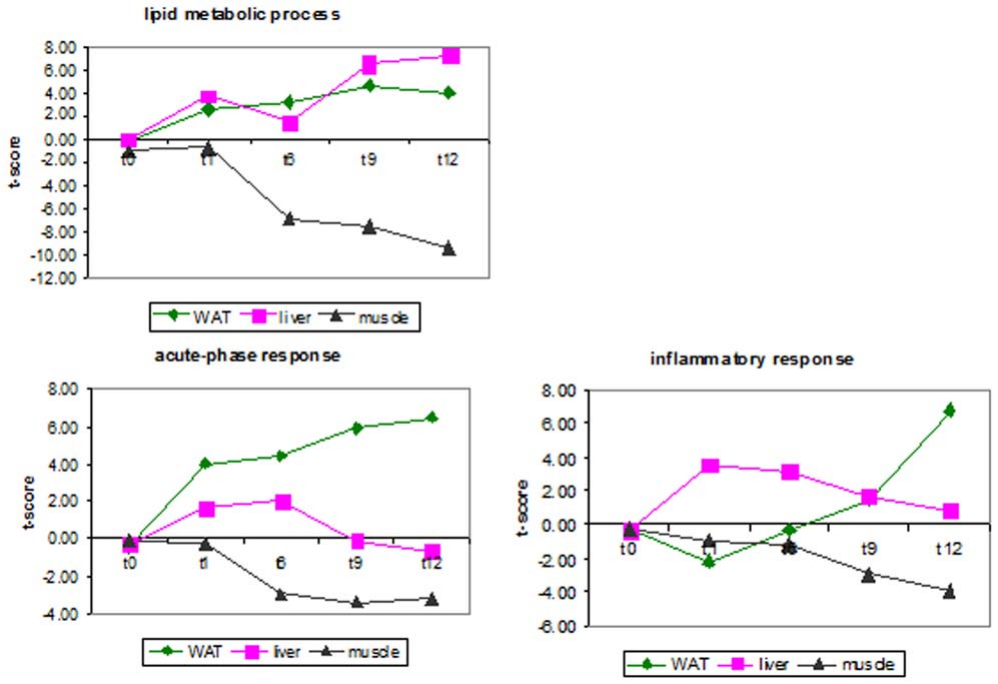
Analysis of biological processes in liver, WAT and muscle over time. Response profiles of specific biological processes are shown together with their average t-scores.

Strikingly, the above functional groups that are up-regulated in liver, are down-regulated in muscle, albeit that the onset is more slowly. This is most clear for lipid metabolism, which is positively affected in liver already in week 1 and down-regulated in muscle from week 6 onward (Figure 6). Specific for WAT, oxidoreductase activity and endoplasmatic reticulum processes were upregulated at all time points while opposite effects were found for liver and skeletal muscle.

### Time-Resolved and Tissue-Specific Microarray Analysis of Inflammatory Processes

**Figure 7:**
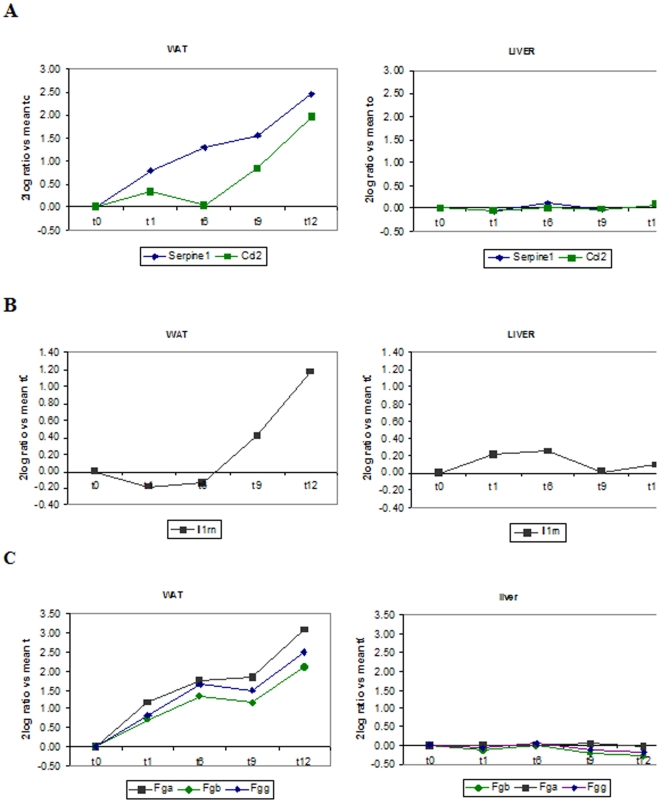
WAT is an important source of inflammation. Comparison of the expression of prototype inflammatory genes in WAT and liver. **A**, PAI-1 (Serpine1) and MCP-1 (Ccl2); **B**, IL-receptor antagonist (Ilm1) and **C**, Fibrinogen-alpha, -beta, -gamma in WAT (left) and liver (right). Data were confirmed in independent RT-PCR analyses.

A distinction also exists between the occurrence of hepatic inflammation and the inflammation that arises in WAT and muscle. Hepatic acute-phase type responses and chronic inflammatory responses were increased as early as one week after starting the HFD feeding, but the acute forms of inflammation completely had returned to control levels in week 9, whereas the chronic inflammatory response decreased continuously, nearing control t = 0 values in week 12 (Figure 6). In WAT, however, the expression of acute-phase response genes increased continuously over time, whereas chronic inflammatory responses (e.g. the genes Ccl2 and interleukin-1 receptor antagonist) increased later (> week 6), reaching highest values in week 12 (Figure 6 and Figure 7, left panel). Direct comparison of WAT and liver revealed that genes encoding for PAI-1, MCP-1 and fibrinogen-α,-β and-γ were predominantly and almost exclusively increased in WAT (Figure 7).

Together, this demonstrates that HFD feeding evokes an acute-type inflammatory response in liver and WAT, and a chronic inflammatory response which declines in liver and increases in WAT over time demonstrating that WAT is an important source of inflammation in the long term disease progression.

**Figure 8:**
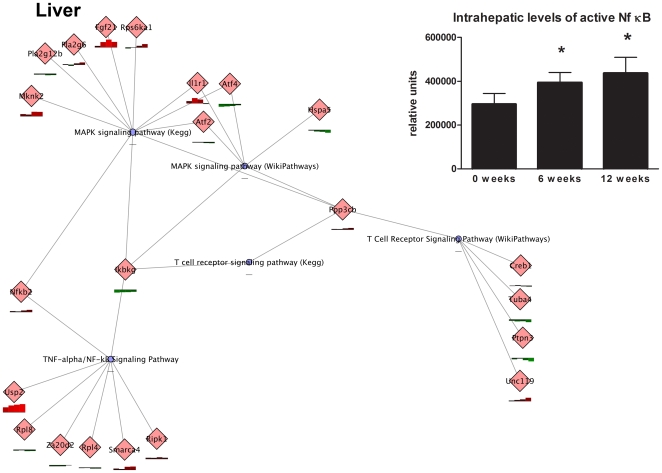
Chronic inflammatory processes in liver modulated by HFD. Transcriptomics data were analyzed by PathVisio with criterion ANOVA qvalue <0.01. Magnifications from the overall networks generated (not shown) illustrating the major inflammatory processes in liver. Red bars indicate upregulated differentially expressed genes, green bars indicated downregulated genes. Blue nodes represent biological processes. The insert shows the amount of active p65-NFkB in liver over time as assessed by TransAM analysis. Data are means ± SD. *P<0.05 compared to t = 0.

**Figure 9:**
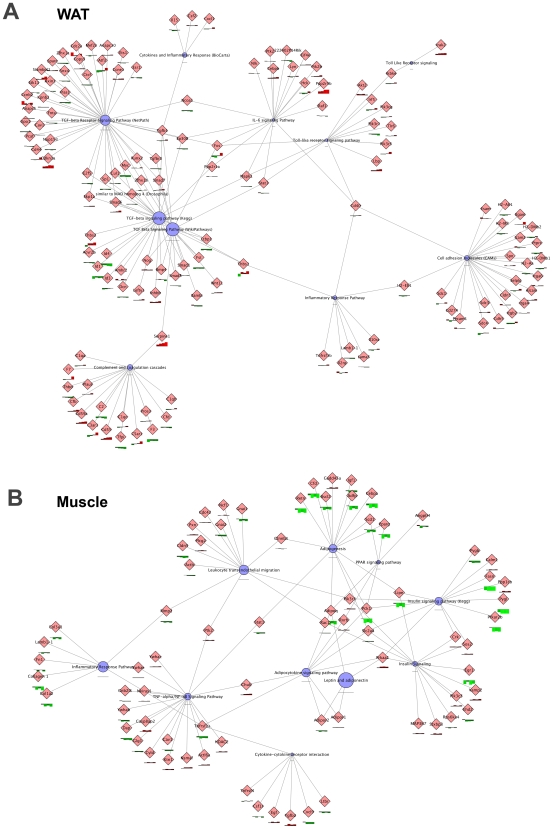
Chronic inflammatory processes in WAT and muscle modulated by HFD. Transcriptomics data were analyzed by PathVisio with criterion ANOVA qvalue <0.01. Magnifications from the overall networks generated (not shown) illustrating the major inflammatory processes in WAT **(A)** as well as the major metabolic processes of skeletal muscle **(B)**. Red bars indicate upregulated differentially expressed genes, green bars indicated downregulated genes. Blue nodes represent biological processes.

In a more refined analysis of the inflammatory processes, PathVisio results were combined with a network representation in Cytoscape. In liver, the TNFalpha/NFkB signaling, MAPK signaling and T cell signaling routes (Figure 8) and, in WAT, the TGFbeta signaling, cell adhesion, Toll-like receptor signaling, complement and coagulation cascades, IL-6 signaling as well as inflammatory cytokine responses (Figure 9A) were stimulated by HFD during the experimental period, indicating that distinct, yet different, inflammatory processes and routes are triggered in the two tissues. Assessment of p65-NFkB activity in liver homogenates by TransAM analysis confirmed an increased activation of this transcription factor over time (Figure 8).

Similarly as seen for the gene expression profiles for carbohydrate- and lipid-related processes, the acute phase and inflammatory responses in muscle are opposite to and also slower in onset than their counterparts in liver (Figures 5 and 6). Consistent with this, among the processes downregulated in muscle are inflammatory response pathways, cytokine receptor interactions, leukocyte transendothelial migration, TNFalpha/NFkB signaling, insulin signaling and adipokine signaling (Figure 9B), clearly demonstrating that HFD feeding suppresses baseline inflammation in skeletal muscle. Analysis of protein expression of transcription factors SOCS-3 and PTP1B, which have been positively associated with IR [10], [33], by Western blotting revealed a significant increase of SOCS-3 in WAT from week 6 onward (Figure S2). SOCS-3 protein levels remained unchanged in liver and skeletal muscle and PTP1B protein expression levels decreased over time in all three tissues.

### Time-Resolved and Tissue-Specific Analysis of Metabolites

To further substantiate the gene expression findings, we next analyzed changes in the concentrations of metabolites reportedly related to IR development within the different tissues.

The occurrence of IR in liver in week 6 was paralleled by an increase in intrahepatic glucose concentrations, which remained high until the end of the study (assessed by GCxGC-MS metabolomics in livers collected under baseline, i.e. non-clamp, conditions; Table 2). Consistent with the rapid adaptations of carbohydrate metabolism seen at the gene level and the reduction of endogenous HGP under baseline conditions (Figure 4B, left panel), the intermediate metabolites of gluconeogenesis were consistently reduced with HFD feeding (Table 2 and Figure S3). Also, metabolites of the Krebs cycle decreased gradually over time (Table 2).

**Table 2:** Intrahepatic metabolites.

	t = 0	6 w	12 w
**Gluconeogenesis**			
Pyrophosphate	100±39	79±30	34±8*
Phosphoenol-PP	100±54	89±41	57±22
Fructose-6-phosphate	100±45	71±32	62±23*
Glucose-6-phosphate	100±29	83±31	54±27*
Glucose	100±28	144±63	187±106*
**Krebs Cycle**			
Succinic acid	100±26	91±25	81±19
Fumaric acid	100±40	52±7*	41±6*
Citric acid	100±27	66±13*	46±9*
**Branched chain amino acids**			
L-Leucine	100±	89±21	69±26*
L-Valine	100±22	86±32	66±18*
L-Isoleucine	100±29	83±10	61±18*
L-Cysteine	100±9	143±31*	112±27

The effect of HFD feeding on intrahepatic metabolites was assessed by GCxGC-MS-based metabolomics analysis using liver tissue biopsies. The same livers that were used for microarray analysis were analyzed. Data are presented as relative changes in metabolite levels over time with t = 0 set 100% and as means ± SD. *P<0.05 compared to t = 0.

The intrahepatic concentration of the branched chain amino acids (BCAA) valine, leucine and isoleucine, the intake of which can improve IR in humans [34], [35], decreased over time (Table 2). By contrast, the intrahepatic concentration of a non-BCAA, cysteine, increased.

**Figure 10:**
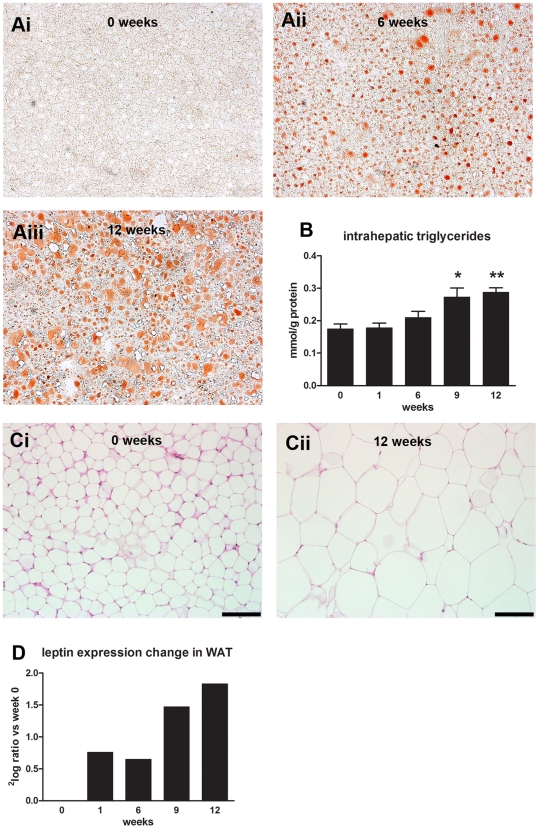
HFD feeding causes hepatosteatosis and WAT hypertrophy. (**A**) Representative photomicrographs of Oil Red O-stained liver cross-sections at t = 0 (i), week 6 (ii) and week 12 (iii). (**B**) Analysis of intrahepatic triglycerides. Values are mean ± SEM *P<0.01 vs t = 0, **P<0.001 vs t = 0. (**C**) HPS-stained epididymal adipose tissue at t = 0 (i) and week 12 (ii). Biopsies from tissues also analyzed by microarray were used. Bars indicate 100 µm. (**D**) Leptin expression over time.

Fatty acid analysis of hepatic homogenates revealed that the observed changes of lipid metabolism genes in the first 6 weeks in liver and WAT were paralleled by pronounced changes of lipid composition in these tissues (Table 3). For example, after 12 w of HFD feeding, oleic acid (C18∶1) was 11.2-fold (P<0.05) increased in liver and 1.6-fold (P<0.05) increased in WAT while α-linolenic acid (C18∶3 n-3) was decreased 2.6-fold (P<0.05) in liver and 8-fold (P<0.05) in WAT (Table S3). Of note, these changes in hepatic lipid composition occurred already during the first 6 weeks of HFD feeding, i.e. before significant elevations in intrahepatic triglyceride concentrations were detected. Figure 10A/B shows that hepatosteatosis became apparent from week 6 onward.

**Table 3:** Intrahepatic saturated, monounsaturated and polyunsaturated fatty acids.

Intrahepatic saturated fatty acids				
	weeks	0	6	12
myristic acid	C14∶0	0.26±0.05	0.72±0.20	1.32±0.47*
palmitic acid	C16∶0	6.0±1.3	12.5±3.8	23.8±9.1
stearic acid	C18∶0	1.7±0.2	2.9±0.3	3.4±0.8*
eicosanoic acid	C20∶0	0.07±0.02	0.13±0.04	0.32±0.10*
behenic acid	C22∶0	0.020±0.004	0.019±0.003	0.040±0.008*
Intrahepatic monounsaturated fatty acids				
	weeks	0	6	12
palmitoleic acid	C16∶1	0.83±0.25	2.27±0.86	4.18±1.61*
oleic acid	C18∶1	3.9±1.2	19.5±7.2	43.5±17.0*
eicosenic acid	C20∶1	0.15±0.5	0.88±0.33	2.62±0.94*
erucic acid	C22∶1	0.02±0.01	0.06±0.02	0.20±0.07*
Intrahepatic polyunsaturated fatty acids n-3 PUFAs				
	weeks	0	6	12
α-linolenic acid	C18∶3 n-3	0.18±0.05	0.06±0.02*	0.07±0.03*
stearidonic acid	C18∶4 n-3	0.013±0.003	0.006±0.002*	0.003±0.001*
eicosatetraenoic acid	C20∶4 n-3	0.060±0.009	0.066±0.007	0.056±0.009
EPA; eicosapentaenoic acid	C20∶5n-3	0.048±0.005	0.043±0.005	0.028±0.005*
DHA; docosahexaenoic acid	C22∶6n-3	0.70±0.12	0.76±0.05	0.63±0.08
Intrahepatic polyunsaturated fatty acids n-6 PUFAs				
	weeks	0	6	12
linoleic acid	C18∶2 n-6	3.47±0.87	1.18±0.56	2.31±0.80
gamma-linolenic acid	C18∶3 n-6	0.09±0.02	0.06±0.02	0.05±0.02
dihomo-γ-linolenic acid	C20∶3 n-6	0.01±0.00	0.42±0.06*	0.57±.013*
arachidonic acid	C20∶4 n-6	0.98±0.16	1.02±0.07	0.87±0.11
adrenic acid	C22∶4 n-6	0.05±0.01	0.06±0.02	0.06±0.01

Quantitative LC/MS analysis of defined fatty acids in tissue homogenates of liver (comparable effects were seen for WAT; not shown). Data are presented as relative units and provided as means ± SEM. *P<0.05 compared to t = 0. Livers of the same animals used for microarray analysis were analyzed.

Parallel with the gene expression changes in WAT (for example, enhanced lipid metabolism and increased expression of adipose differentiation related protein, ADRP, not shown), morphological analysis of WAT revealed a pronounced increase in adipocyte size (hypertrophy; Figure 10C). In addition, leptin mRNA levels in WAT were strongly increased (Figure 10D), indicating that not merely the increase in fat mass but also enhanced gene expression is responsible for the elevated leptin levels in plasma.

## Discussion

**Figure 11:**
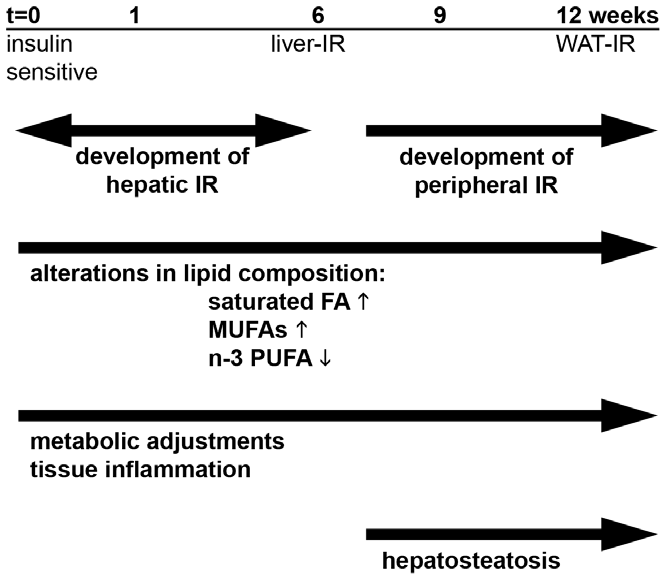
Overview of development of IR in ApoE3Leiden mice fed a HFD. Weeks of HFD feeding are indicated together with the time point at which liver, and WAT developed IR. Changes in the lipid composition (ie. the quality and nature of fatty acids) occurred within the first 6 weeks and changes persisted until the end of the study. Extensive metabolic adjustments (indicating metabolic stress) and development of local inflammation in liver and WAT from week 1 onward. Hepatosteatosis developed after week 6.

The sequence of events leading to the development of IR as well as the risk factors and regulatory mechanisms contributing to its pathogenesis are incompletely understood. This may be due, at least in part, to the limitations of the animal models typically used [36], but also to a lack of integrative, time-resolved approaches to understand the complexity of the disease. In the present study we used E3L mice, which exhibit a humanized lipoprotein metabolism [11], [37], and treated these mice with a HF diet for 12 weeks. During this period, animals became increasingly obese (with greatest increases in adiposity occurring after week 6), and developed a gradual increase in glucose intolerance (by GTT) and HOMA. Radioactive clamp analysis revealed decreased hepatic insulin sensitivity after 6 weeks, while WAT (but not muscle) became insulin resistant after 12 weeks, i.e. notably later than liver (Figure 11). In a systems biology approach, we have clarified the time-course and tissue-specificity of the underlying metabolic adaptations (viz. carbohydrate and lipid metabolism), pathologic consequences (e.g. change from beneficial polyunsaturated fatty acids to unfavourable saturated fatty acids), as well as acute and chronic inflammatory responses.

Microarray data provide solid evidence in support of a sequence of time- and tissue-specific events during the development of IR after starting the HF diet. The liver acutely responds by up-regulating the expression of genes involved in glucose metabolic processes, (cellular) carbohydrate metabolic processes, fatty acid biosynthetic and metabolic processes, (cellular) lipid metabolic processes, and lipid transport, while in WAT only the expression of genes involved in (cellular) lipid metabolic processes and lipid transport increase. Strikingly, the above upregulated functional groups in liver are all down-regulated in muscle, albeit with a slower onset.

The rapid metabolic adaptations in liver in the first week after starting HFD (which did not affect plasma glucose or insulin levels) coincide with an impaired ability to clear a bolus of glucose as demonstrated in a glucose tolerance test. This phenomenon has also been described by others [38], yet little is known about the impact and pathologic consequences of this ‘acute’ metabolic response.

Free fatty acid analysis further detailed the nature of the metabolic adaptations as revealed by transcriptomics. Our data clearly show that the content of specific IR-associated [39] saturated and monounsaturated fatty acids increases in liver and WAT, while, at the same time, the content of PUFAs with beneficial effect on IR [40] decreases. Interestingly, these tissue-specific effects were reflected by similar changes in lipid composition in plasma. These changes cannot be detected with standard clinical chemistry or routine lipid analyses and may therefore have gone unnoticed in previous studies investigating diet-induced obesity and IR. To our knowledge, our data demonstrate for the first time and comprehensively that a change of lipid composition in WAT and liver (and reflected in plasma) coincide with the development of IR in these tissues. Our findings also provide support to findings in humans indicating that specific fatty acids are participating in the pathogenesis of IR, and that the ratio of saturated to unsaturated fatty acids may be an important determining factor for IR, independent of abdominal obesity [39], [41].

In humans, whole body IR depends to a great extent on muscle IR. In the present study, skeletal muscle did not become insulin resistant during 12 weeks of HFD feeding. There are important differences between humans and mice regarding glucose metabolism in muscle. For example, the CHC22 clathrin heavy-chain isoform is highly expressed in human skeletal muscle and plays a key role in glucose metabolism whereas mice only express a pseudogene for this protein [42]. Tissue-specific introduction of CHC22 in mice caused aberrant localization of GLUT4 transport pathway components in their muscle, as well as more human-like features of diabetes [42].

The plasma concentrations of NEFA were not markedly affected upon HFD feeding. Development of IR is associated with elevated plasma levels of NEFA and NEFA have long been recognized for their contribution to decreasing insulin-mediated glucose disposal [43]. In C57BL/6 mice (and thus also ApoE3Leiden mice) however, NEFA typically decrease during high fat diet feeding and this discrepancy from the human situation has also been reported by others [44], [45]. The underlying molecular cause is not fully understood. The effect may be related to a recent observation of Oosterveer *et al*.[45] who demonstrated that HFD feeding lowers VLDL production and decreases the relative triglyceride content of VLDL particles in C57BL/6 mice.

There is some controversy about whether hepatic IR induces lipid accumulation in liver (hepatosteatosis) or whether hepatosteatosis itself is a requirement for the development of hepatic IR [46], [47]. Our finding that significant accumulation of intrahepatic triglycerides occurs after the establishment of IR lends support to the concept that hepatic IR precedes hepatosteatosis rather than to the view that hepatosteatosis is a requirement for the development of hepatic IR.

Our finding that the induction of IR by HFD is accompanied by chronic, low-grade inflammation, as reflected by increased plasma levels of the inflammation markers SAA, fibrinogen, VCAM-1 and E-selectin, is in line with the notion that inflammation is crucial for the development of IR [48]. Epidemiologists have consistently found that chronically elevated plasma levels of systemic markers and potential mediators of inflammation and the acute-phase response such as C-reactive protein, fibrinogen, interleukin 6, tumor necrosis factor-alpha (TNFα), and macrophage migration inhibitory factor (MIF) are associated with an increased risk of future IR [4], [49], suggesting that factors that either elicit or contribute to chronic inflammation may participate in the pathogenesis of IR and DM2. Indeed, proinflammatory cytokines and acute-phase reactants have been reported to be implicated in the development of IR in humans [3], [50] and in rodents [5], [51], [52]. The biological relevance of the activation of inflammatory pathways in IR development is further underscored by the demonstration that interference with these pathways can improve or alleviate IR [3], [5], [6], [8].

Our transcriptomics analysis adds further important new information to the role of inflammation in the pathogenesis of IR by specifying the time-course, tissue-specificity and nature of the inflammatory component. This analysis shows that HFD feeding evokes an acute-phase and a (chronic) inflammatory response in liver and WAT, but suppresses the inflammatory state of muscle. Importantly, the inflammatory responses in liver and WAT differ in time-course as well as in the nature of the inflammatory components. Both the acute-phase and the inflammatory response are mainly transient in liver, while the inflammatory responses in WAT increase over time, indicating that WAT is an important source of inflammation in the long run.

In liver, the NFkB signaling route is moderately activated by HFD feeding as identified by network analysis. Using a functional p65-NFkB-binding assay (TransAM) we showed an increase in NF-kB activity during HFD feeding in week 6, i.e. the time point at which the liver became insulin resistant. This observations is in agreement with data from others showing that already slight elevations in hepatic NF-kB activity can cause liver IR [8]. We also found an increase in SOCS-3 protein expression in WAT from week 6 onward. This effect may contribute to IR in WAT as overexpression of SOCS-3 in adipose tissue [53], liver [10] or skeletal muscle [54] caused local IR.

The chronic inflammatory response of WAT involves an increased expression of the genes encoding for interleukin-1 receptor antagonist, CCL2/MCP-1 and PAI-1. Others have shown that interleukin-1 receptor antagonist is overexpressed in WAT of obese humans [55] and that CCL2/MCP-1 contributes to adipose IR and the development hepatic steatosis [6]. Indeed, in the present study, the development of WAT-specific IR and hepatosteatosis parallels the expression of Ccl2/Mcp-1. The pronounced increase in PAI-1 gene expression may be explained by the observation that the expression of Ppar-gamma, a negative regulator of Pai-1 transcription [56] is reduced by HFD feeding.

Remarkably, comparison of liver and WAT revealed that genes encoding for SAA, PAI-1, MCP-1 and fibrinogen-α,-β and-γ were predominantly and almost exclusively increased in WAT. The plasma inflammation markers, SAA and fibrinogen, are generally viewed as being biosynthesized in liver [57]. Our observation that the concentrations of SAA and fibrinogen are gradually and continuously increasing over the 12-week HFD feeding period would suggest that ‘subacute *hepatic* inflammation’ might be involved in the onset and development of IR. However, contrary to existing views, transcriptomics analysis indicated that the increased SAA and fibrinogen expression occurred in WAT, and not liver. While adipose expression of SAA is well-established [58], fibrinogen α, β and γ gene expression in WAT has not been demonstrated so far.

Together, the present study uses an integrative approach to gain insight into the tissue-specificity and time-course of metabolic and inflammatory processes that underlie the development of HFD-induced IR in a humanized mouse model, E3L mice. Notably, the observed effects can mainly be ascribed to HFD feeding rather than to aging as with chow feeding alone no inflammation or insulin resistance develops within a period of 12 weeks [59]. The data show that both liver and WAT rapidly respond to HFD by metabolic adaptations and inflammatory responses that lead to IR, first in liver and subsequently also in WAT. The biochemical pathways affected are numerous and different for the various tissues, indicating that the design of effective treatment regimens should be at an integral level rather than directed at a single target.

## Supporting Information

Table S1 Diet composition of the high-fat diet used.
(0.10 MB PDF)

Table S2 Saturated, monounsaturated, and polyunsaturated fatty acids in plasma.
(0.06 MB PDF)

Table S3 Saturated, monounsaturated, and polyunsaturated fatty acids in white adipose tissue.
(0.06 MB PDF)

Figure S1 Targeted analysis of genes in pathways. The gene expression changes of glycolysis and gluconeogenesis genes are shown for liver, muscle, and white adipose tissue. Individual gene expression was statistically compared to the expression level at t  = 0, and coloring indicates fold change and significance (green  =  significant in ANOVA; P≤.01).
(0.41 MB PDF)

Figure S2 Western blot analysis of SOCS-3 and PTP1B protein expression in liver, white adipose tissue, and muscle over time. *P<0.05 compared to t  = 0.
(0.03 MB PDF)

Figure S3 Metabolites of gluconeogenesis.
(0.37 MB TIF)
