# Supplementary material for: Time-Resolved and Tissue-Specific Systems Analysis of the Pathogenesis of Insulin Resistance
Source: PLoS One. 2010 Jan 21;5(1):e8817. doi: 10.1371/journal.pone.0008817 (PMC2809107; doi:10.1371/journal.pone.0008817)
Supplement: Table S1 — Diet composition of the high-fat diet used. (0.10 MB PDF) [file pone.0008817.s001.pdf]

4031,05

Reference diet / high fat (rundvet)

**Samenstelling**		**Aandeel**
8500	STAND.VIT.PREMIX	0,30
8503	ST.SPOR.PREMIX	0,30
7559	CaHPO4.2H2O (LAAG F)	1,60
7560	CaCO3 REINST/Me.2069	1,20
7547	KH2PO4	0,87
7546	KCl.	0,87
7083	ZOUT (GEZAKT).	0,37
7611	MgSO4.7H2O	0,50
7552	MgO SCHWER REINST	0,25
7082	METHIONINE SYNTH. DL	0,30
7514	CHOLINE CL 50%	0,40
7599	ZURE CASEINE.	24,00
7579	MAISZETMEEL GEL INST	18,67
7584	DICACEL2+4/cellulose	6,00
7519	RUNDVET	24,00
7060	CERELOSE/dextrose	20,38
		**100,00**

**Analyse**		**Eenheid**	**Aandeel**
1	Cr.Prot	g/kg	214,41
2	Cr. Fat	g/kg	240,00
3	Cr.Fiber	g/kg	61,57
4	Minerals	g/kg	22,45
5	Moisture	g/kg	57,65
6	Sug.+St.	g/kg	350,46
7	Nfree ex	g/kg	368,47
8	Dry Mat.	g/kg	938,87
9	Lysine	g/kg	14,90
10	Methion.	g/kg	8,73
12	Cystine	g/kg	0,62
13	Threonin	g/kg	8,54
14	Tryptoph	g/kg	2,78
15	Isoleuc.	g/kg	12,10
16	Arginine	g/kg	7,34
17	Phenylal	g/kg	8,74
18	Histidin	g/kg	5,57
19	Leucine	g/kg	22,85
20	Tyrosine	g/kg	11,33
21	Valine	g/kg	14,11
22	Alanine	g/kg	4,75
23	Asp.acid	g/kg	11,11
24	Glut.ac.	g/kg	42,12
25	Glycine	g/kg	6,14
26	Proline	g/kg	19,27
28	Serine	g/kg	6,74
30	Calcium	g/kg	8,63
31	Phos.tot	g/kg	5,17
34	Potass.	g/kg	7,04
35	Magnes.	g/kg	1,96
36	Sodium	g/kg	1,41
37	Chlorine	g/kg	6,73
38	Sulfur	g/kg	0,68

49	C8-C12:0	g/kg	0,72
50	C14:0	g/kg	8,16
51	C16:0	g/kg	61,68
52	C16:1	g/kg	7,68
53	C18:0	g/kg	50,88
54	C18:1	g/kg	88,80
55	C18:2	g/kg	7,68
56	C18:3	g/kg	1,92
57	C20-C22	g/kg	2,64
61	Vit. A	IU/g	21,60
63	Vit. D	IU/g	2,40
64	Vit. D3	IU/g	2,40
65	Vit. E	mg.	75,20
67	Vit. K3	mg.	12,00
68	Vit. B1	mg.	24,00
69	Vit. B2	mg.	13,87
70	Vit. B6	mg.	18,40
71	Niacin	mg.	47,04
72	Pant.ac.	mg.	19,08
73	Vit.B12	mcg.	60,00
74	Folic.ac	mg.	9,41
75	Choline	mg.	1.491,56
76	Biotin	mcg.	367,98
77	Inositol	mg.	599,97
82	Starch	g/kg	146,53
83	Sugars	g/kg	185,97
86	Lactose	g/kg	0,48
91	Cellulos	g/kg	54,00
93	Glucose	g/kg	189,93
96	Iron	mg.	154,73
97	Mangan.	mg.	76,20
98	Zinc	mg.	62,89
99	Copper	mg.	21,16
100	Cobalt	mg.	0,17
101	Iodine	mg.	0,57
102	Selenium	mg.	0,22
104	Chromium	mg.	0,59
105	Nickel	mg.	0,08
107	Fluorine	mg.	2,57
109	Arsenic	mg.	0,08
111	Lead	mg.	0,96
113	Alumin.	mg.	3,97
