# Supplementary material for: Time-Resolved and Tissue-Specific Systems Analysis of the Pathogenesis of Insulin Resistance
Source: PLoS One. 2010 Jan 21;5(1):e8817. doi: 10.1371/journal.pone.0008817 (PMC2809107; doi:10.1371/journal.pone.0008817)
Supplement: Table S2 — Saturated, monounsaturated, and polyunsaturated fatty acids in plasma. (0.06 MB PDF) [file pone.0008817.s002.pdf]

Table S2. Saturated, monounsaturated and polyunsaturated fatty acids in plasma.

Circulating saturated fatty acids

	weeks	0	12
palmitic acid	C16:0	10.6 ± 0.6	10.4 ± 0.4
stearic acid	C18:0	2.98 ± 0.15	4.58 ± 0.17*
eicosanoic acid	C20:0	0.09 ± 0.01	0.08 ± 0.01
behenic acid	C22:0	0.039 ± 0.002	0.023 ± 0.003*

Circulating monounsaturated fatty acids

	weeks	0	12
palmitoleic acid	C16:1	4.32 ± 0.64	3.60 ± 0.21
oleic acid	C18:1	10.08 ± 0.60	16.23 ± 0.70*
eicosenic acid	C20:1	0.34 ± 0.04	0.57 ± 0.03*
erucic acid	C22:1	0.033 ± 0.004	0.035 ± 0.002

Circulating polyunsaturated fatty acids n-3 PUFAs

	weeks	0	12
α-linolenic acid	C18:3 n-3	0.668 ± 0.053	0.072 ± 0.005*
stearidonic acid	C18:4 n-3	0.035 ± 0.006	0.001 ± 0.000*
eicosatetraenoic acid	C20:4 n-3	0.072 ± 0.009	0.054 ± 0.004
EPA; eicosapentaenoic acid	C20:5n-3	0.113 ± 0.025	0.040 ± 0.002*
DHA; docosahexaenoic acid	C22:6n-3	0.63 ± 0.08	0.65 ± 0.04

Circulating polyunsaturated fatty acids n-6 PUFAs

	weeks	0	12
linoleic acid	C18:2 n-6	7.70 ± 0.30	1.83 ± 0.12*
gamma-linolenic acid	C18:3 n-6	0.201 ± 0.020	0.035 ± 0.002*
dihomo-γ-linolenic acid	C20:3 n-6	0.032 ± 0.004	0.580 ± 0.035*
arachidonic acid	C20:4 n-6	1.04 ± 0.10	0.98 ± 0.07
adrenic acid	C22:4 n-6	0.088 ± 0.007	0.032 ± 0.002*

Quantitative, LC/MS-based lipidomics analysis of defined fatty acids in plasma over time. Plasma of the same animals used for microarray analysis were used. Data presented are relative units and provided as means±SEM. *P<0.05 compared to t=0.
