# Supplementary material for: Time-Resolved and Tissue-Specific Systems Analysis of the Pathogenesis of Insulin Resistance
Source: PLoS One. 2010 Jan 21;5(1):e8817. doi: 10.1371/journal.pone.0008817 (PMC2809107; doi:10.1371/journal.pone.0008817)
Supplement: Table S3 — Saturated, monounsaturated, and polyunsaturated fatty acids in white adipose tissue. (0.06 MB PDF) [file pone.0008817.s003.pdf]

Table S3. Saturated, monounsaturated and polyunsaturated fatty acids in white adipose tissue.

Intra-WAT saturated fatty acids

	weeks	0	12
myristic acid	C14:0	0.38±0.05	0.47±0.04
palmitic acid	C16:0	2.99±0.18	3.14 ± 0.30
stearic acid	C18:0	0.52±0.01	0.72 ± 0.05*
eicosanoic acid	C20:0	0.017 ± 0.004	0.015 ± 0.001

Intra-WAT monounsaturated fatty acids

	weeks	0	12
palmitoleic acid	C16:1	1.43 ± 0.19	1.58 ± 0.18
oleic acid	C18:1	4.00± 0.32	6.46 ± 0.71*
eicosenic acid	C20:1	0.002 ± 0.001	0.003 ± 0.001*
erucic acid	C22:1	0.006 ± 0.001	0.004 ± 0.001

Intra-WAT polyunsaturated fatty acids n-3 PUFAs

	weeks	0	12
α-linolenic acid	C18:3 n-3	0.279 ± 0.030	0.035 ± 0.005*
stearidonic acid	C18:4 n-3	0.0011 ± 0.0005	0.0001± 0.0001*
eicosatetraenoic acid	C20:4 n-3	0.004 ± 0.001	0.002 ± 0.001*
EPA; eicosapentaenoic acid	C20:5n-3	0.007 ± 0.001	0.003 ± 0.001*
DHA; docosahexaenoic acid	C22:6n-3	0.023 ± 0.003	0.009 ± 0.001*

Intra-WAT polyunsaturated fatty acids n-6 PUFAs

	weeks	0	12
linoleic acid	C18:2 n-6	3.50 ± 0.30	0.72 ± 0.09*
gamma-linolenic acid	C18:3 n-6	0.024 ± 0.003	0.004 ± 0.001*
dihomo-γ-linolenic acid	C20:3 n-6	0.004 ± 0.001	0.020 ± 0.002*
arachidonic acid	C20:4 n-6	0.066 ± 0.007	0.025 ± 0.008*
adrenic acid	C22:4 n-6	0.006 ± 0.001	0.003 ± 0.001*

Quantitative, LC/MS-based lipidomics analysis of defined fatty acids in WAT over time. WAT of the same animals used for microarray analysis were used. Data presented are relative units and provided as means±SEM. *P<0.05 compared to t=0.
