# Supplementary material for: Time-Resolved and Tissue-Specific Systems Analysis of the Pathogenesis of Insulin Resistance
Source: PLoS One. 2010 Jan 21;5(1):e8817. doi: 10.1371/journal.pone.0008817 (PMC2809107; doi:10.1371/journal.pone.0008817)

Figure S1

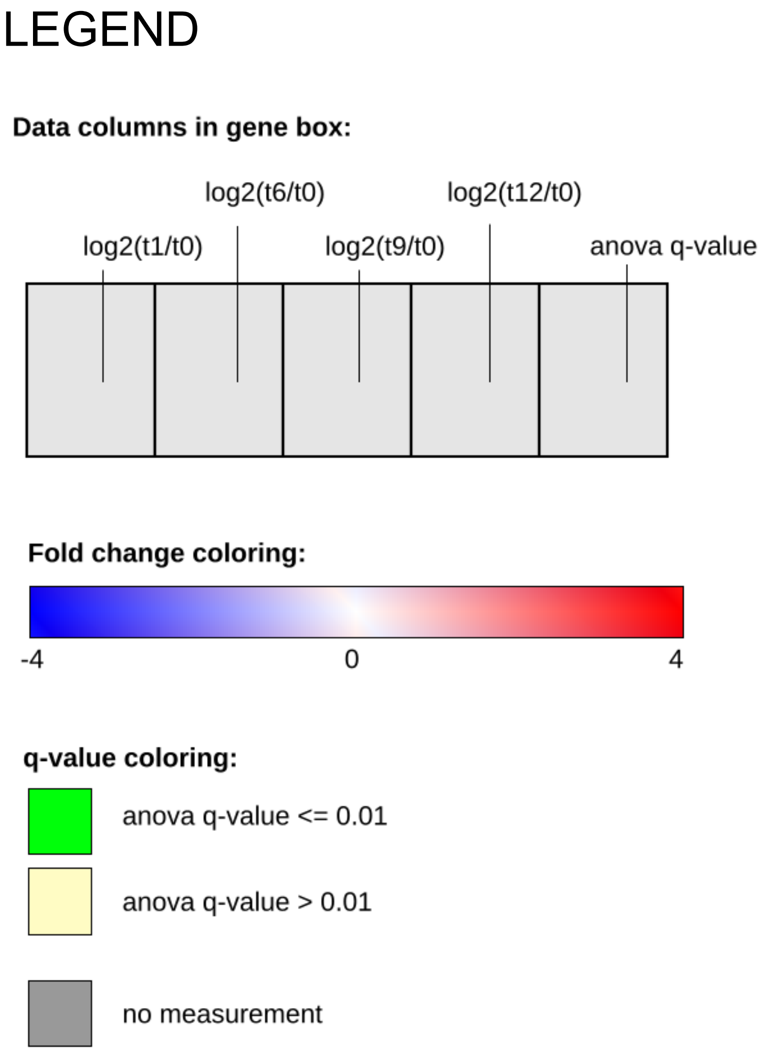
LEGEND
Data columns in gene box:
log2(t6/t0)
log2(t12/t0)
log2(t1/t0)
log2(t9/t0)
anova q-value
Fold change coloring:
-4
0
4
q-value coloring:
anova q-value <= 0.01
anova q-value > 0.01
no measurement

Title: Glycolysis and Gluconeogenesis
Email: genmapp@gladstone.ucsf.edu
Availability: 2000, Gladstone Institutes
Last modified: 12/04/2003
Organism: Mus musculus
Data Source: GenMAPP 2.0

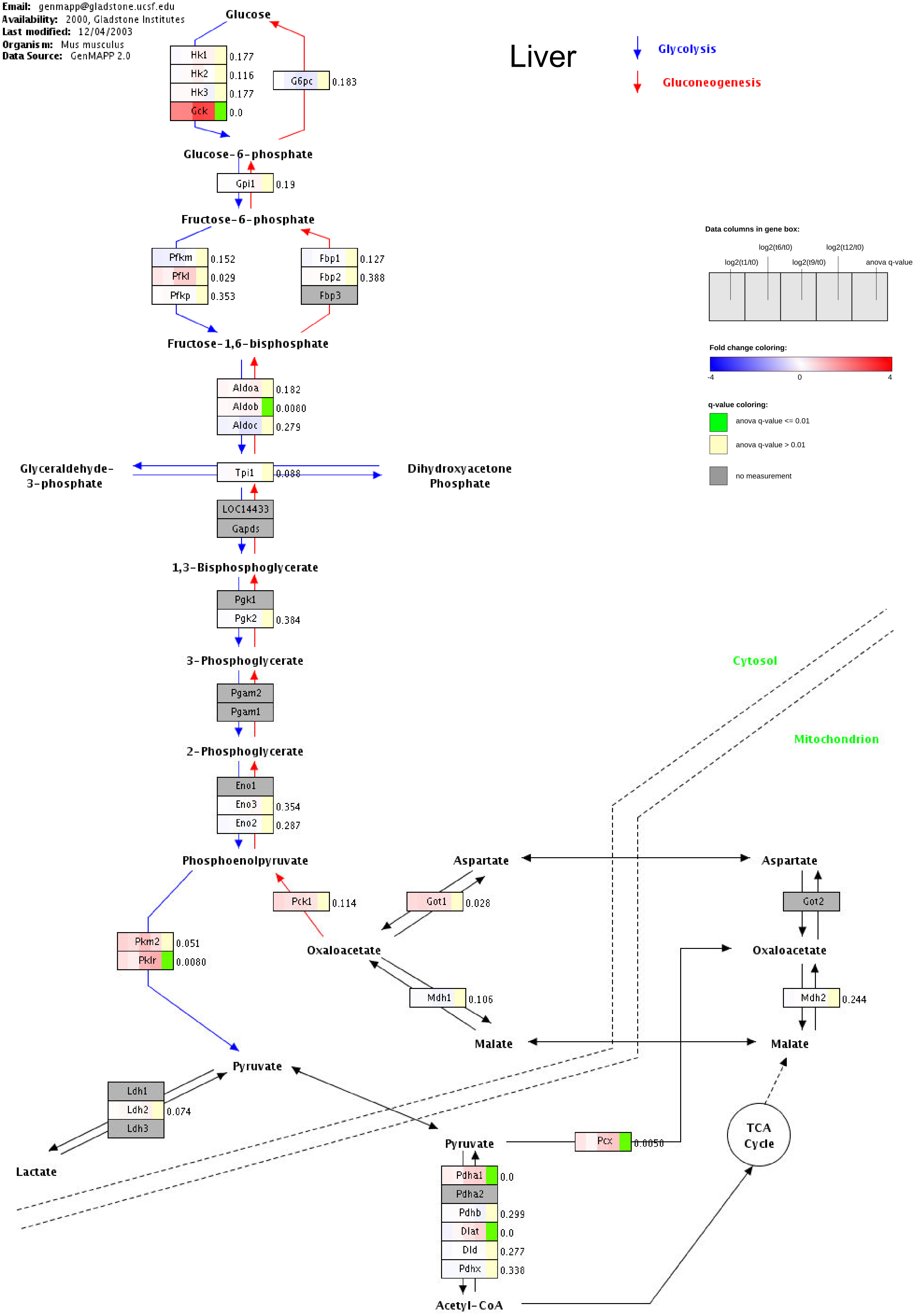

Title: Glycolysis and Gluconeogenesis
Email: genmapp@gladstone.ucsf.edu
Availability: 2000, Gladstone Institutes
Last modified: 12/04/2003
Organism: Mus musculus
Data Source: GenMAPP 2.0

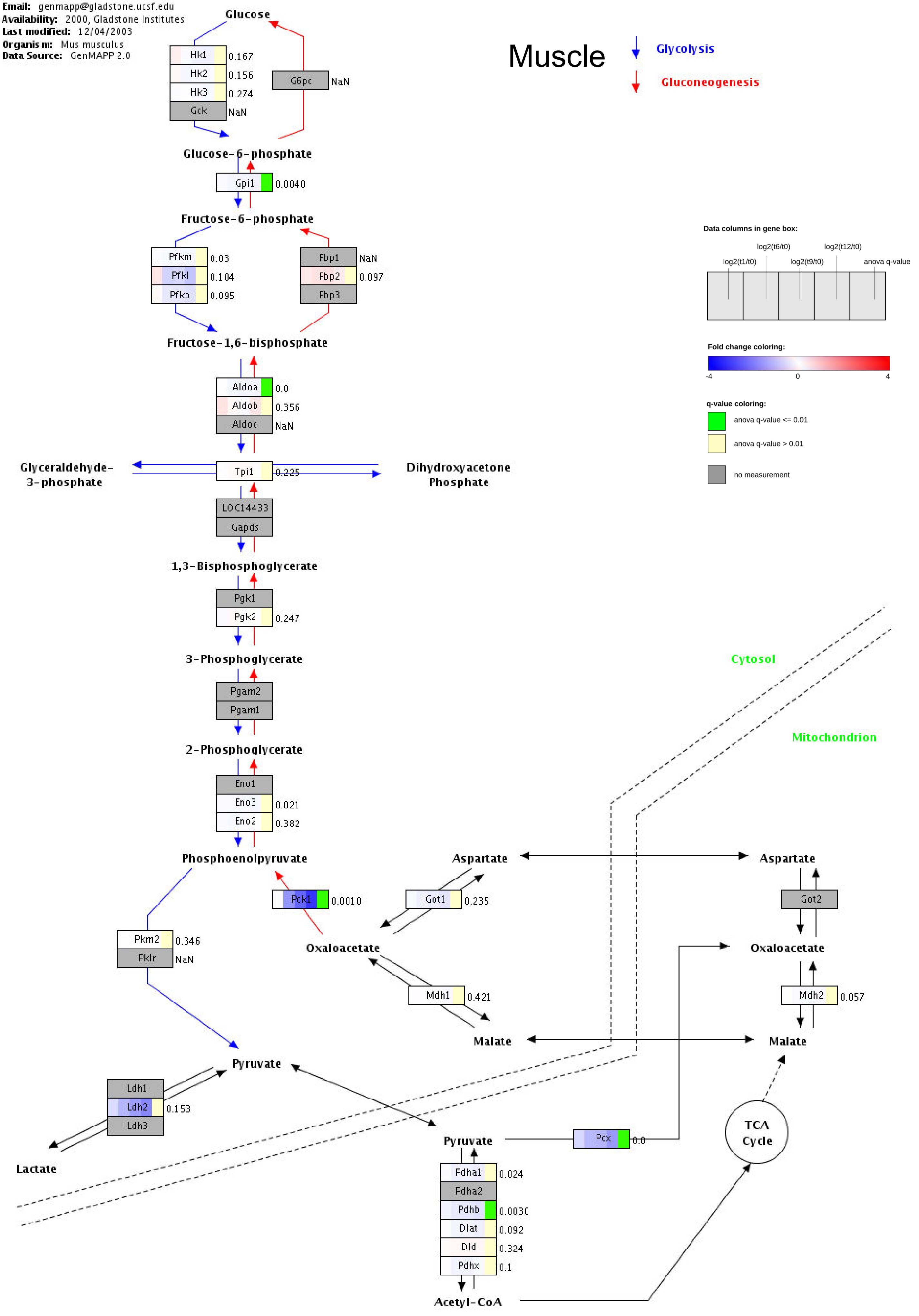

Title: Glycolysis and Gluconeogenesis
Email: genmapp@gladstone.ucsf.edu
Availability: 2000, Gladstone Institutes
Last modified: 12/04/2003
Organism: Mus musculus
Data Source: GenMAPP 2.0

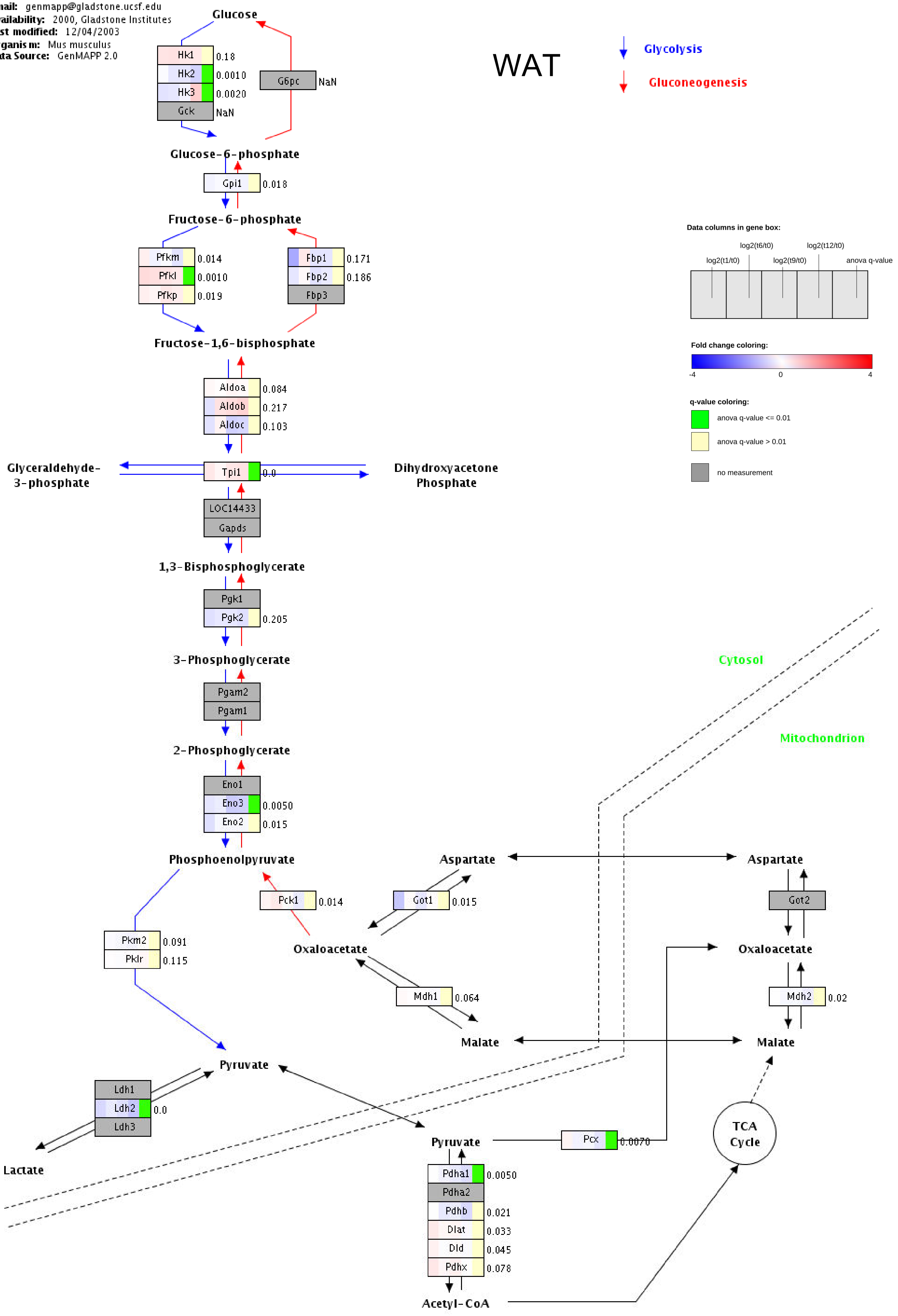

Supplement: Figure S1 — Targeted analysis of genes in pathways. The gene expression changes of glycolysis and gluconeogenesis genes are shown for liver, muscle, and white adipose tissue. Individual gene expression was statistically compared to the expression level at t = 0, and coloring indicates fold change and significance (green = significant in ANOVA; P≤.01). (0.41 MB PDF) [file pone.0008817.s004.pdf]
