# Supplementary figures and images for: Time-Resolved and Tissue-Specific Systems Analysis of the Pathogenesis of Insulin Resistance
Source: PLoS One. 2010 Jan 21;5(1):e8817. doi: 10.1371/journal.pone.0008817 (PMC2809107; doi:10.1371/journal.pone.0008817)

Figure S2

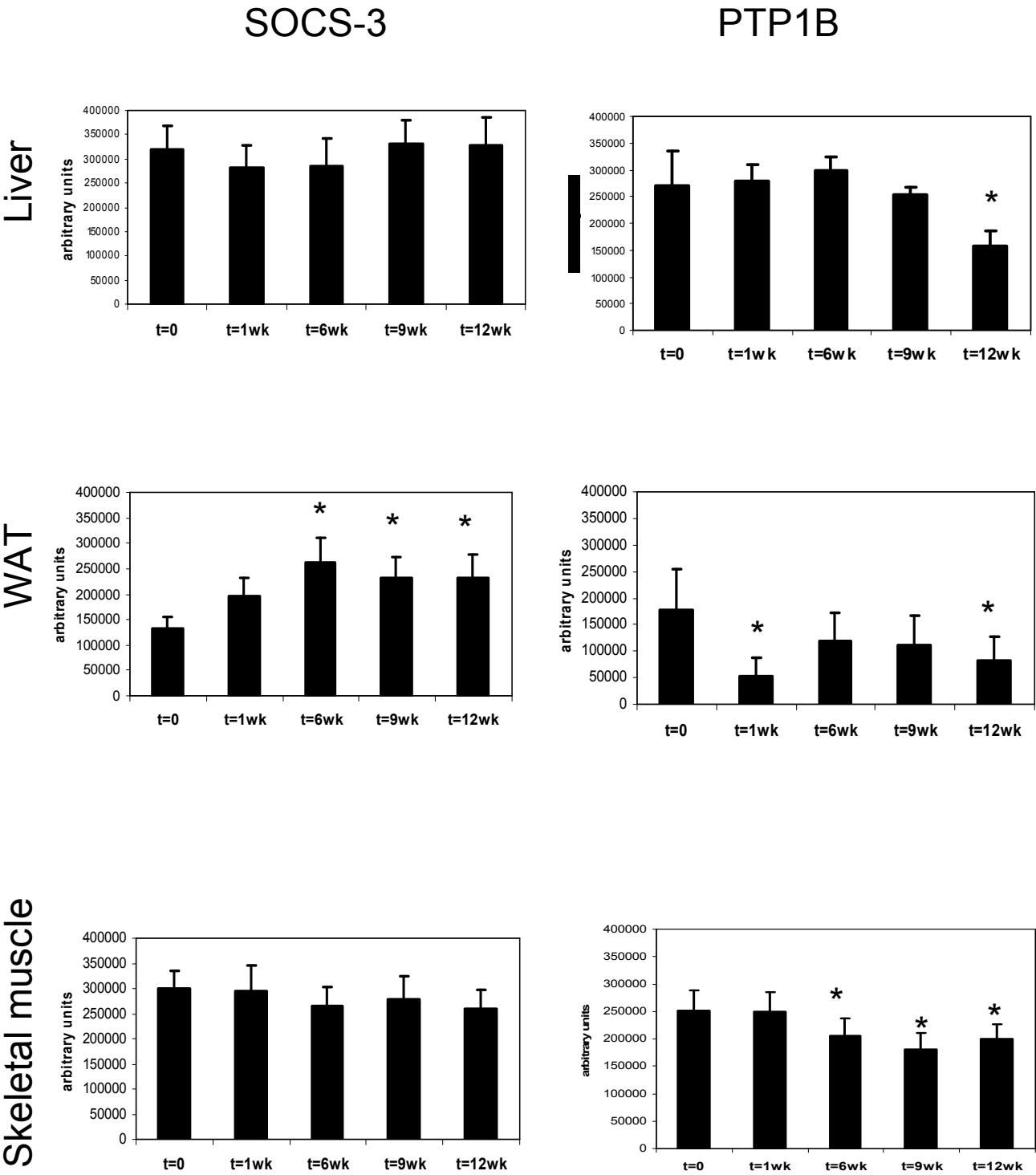

Supplement: Figure S2 — Western blot analysis of SOCS-3 and PTP1B protein expression in liver, white adipose tissue, and muscle over time. *P<0.05 compared to t = 0. (0.03 MB PDF) [file pone.0008817.s005.pdf]

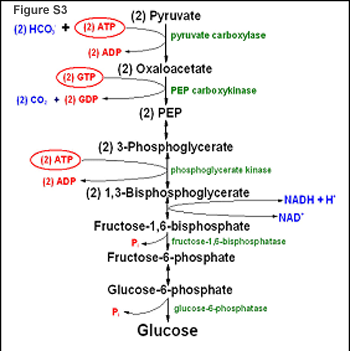

Supplement: Figure S3 — Metabolites of gluconeogenesis. (0.37 MB TIF) [file pone.0008817.s006.tif]
